# Fiddler on the Tree - A Bush-Cricket Species with Unusual Stridulatory Organs and Song

**DOI:** 10.1371/journal.pone.0092366

**Published:** 2014-03-18

**Authors:** Klaus-Gerhard Heller, Claudia Hemp

**Affiliations:** 1 Department of Biology, Humboldt-Universität zu Berlin, Berlin, Germany; 2 Department of Animal Ecology and Tropical Biology, University of Würzburg, Würzburg, Germany; Universite Paris XI – CNRS, France

## Abstract

Insects of the order Orthoptera are well-known for their acoustic communication. The structures used for this purpose show a high diversity which obviously relates to differences in song parameters and to the physics of sound production. Here we describe song and morphology of the sound producing organs of a tropical bush-cricket, *Ectomoptera nepicauda*, from East Africa. It has a very unusual calling song consisting of frequency-modulated, pure-tone sounds in the high ultrasonic range of 80 to120 kHz and produced by extremely fast wing movements. Concerning morphology, it represents the most extreme state in the degree of left-right fore-wing differentiation found among Orthoptera: the acoustic parts of the left fore-wing consist exclusively of the stridulatory file, comparable in function to the bow of a violin, while the right wing carries only the plectrum ( =  string) and mirror ( =  soundbox).

## Introduction

Orthopteran insects belonging to the suborder Ensifera (crickets, mole-crickets, bush-crickets/katydids) typically stridulate by rubbing their fore-wings against each other. Only some small groups, which have probably lost these primary tegminal stridulatory organs [1], use other mechanisms. The wings contain a file-and-scraper-system consisting of series of sclerotized teeth on a vein on the underside of the one wing (file), while the scraper is formed by a sclerotized ridge at the edge of the other wing. For songs transmitted over distance the wings also have to contain resonating elements for amplification. In crickets (Gryllodea) the most prominent structures used in this context are called harp and mirror (e.g., [2]). In this group left and right fore-wings are typically quite similar with stridulatory files and harps on both sides. However, the different groups have adopted different wing overlaps. ‘True’ crickets usually sing right over left wing, using the file of the right wing [3]. Mole crickets (Gryllotalpidae) and Prophalangopsidae seem to be able to switch between the wings for stridulation (e.g., [4]), perhaps an expression of an ancestral condition. To produce sound efficiently, always both harps must vibrate in phase with structures near the plectrum acting as a phase shifter [5], [6] resulting in a tonal song dominated by one carrier frequency.

In bush-crickets ( = katydids) the situation is different. Here only the file of the upper left tegmen is used and that of the right one is only vestigial (see [7], [8] for very rare exceptions). According to indirect experiments [9], [10] and recent measurements [11] also the function of both tegmina is different, at least in some groups. There seems to be some kind of specialisation: the left tegmen carries the file, the right the scraper (plectrum) and resonating structures.

Besides carrying the functional file, the left tegmen appears to provide some unavoidable damping resulting in a relatively low quality factor Q and a broad spectrum [11], [12]. It is not yet clear how far these findings can be generalised (see Discussion). Many bush-crickets have indeed much broader spectra than crickets, but there are also species with tonal songs [13].

Broad-banded, non-resonant songs are very common among members of the (sub)family Phaneropterinae(-idae), the False or Bush katydids. In this group only very few species with narrow-banded, tonal songs are known (for a review see [14]). The maximum of the carrier frequencies is usually situated in the audio or low ultrasonic range, and only a few flightless species communicate with broad-banded, purely ultrasonic songs. Phaneropterinae show also a high degree in left-right wing differentiation: together with Pseudophyllinae it is the only group where at least in some species the right wing does not carry any remains of a stridulatory file, as can be concluded from the few studies on this character [15], [16]. However, the different specialisation of the fore-wings of both body sides obviously does not exclude the possibility for the evolution of pure tone songs.

Here we describe morphological structures and the song of a long-winged species, *Ectomoptera nepicauda* Ragge, with extremely asymmetric acoustic parts of the tegmina. On the left tegmen the acoustical structures nearly exclusively consist of the stridulatory file thus reminding of a violin's bow, while on the right mainly plectrum and a large mirror are present. Using this ‘equipment’, the animals emit short, frequency-modulated, tonal sounds between 80 and 120 kHz. Such short signals may be produced either by contact of single teeth of the file with the scraper activating a resonator or by very fast stridulatory movements. Analysing the structure of sound and stridulatory organs we postulate the second mechanism is used for the production of these signals.

## Materials and Methods

### Ethics Statement

All necessary permits were obtained for the described study, which complied with all relevant regulations. We thank the Tanzanian Commission for Science and Technology for permitting research (research permit No 2012-417-ER-96-44).

The genus *Ectomoptera* is restricted to a small area of southern Kenya and north-eastern Tanzania in East Africa. At present one species, *E. nepicauda*, is described while several still undescribed species occur in the area [17]. All species are forest bound canopy dwellers in lowland and submontane forest in the East Usambara Mountains, the Shimba Hills and coastal forest. The animals (three males and three females) were collected in the East Usambara Mountains of north-eastern Tanzania, in March 2012 along the Zigi Trail in lowland evergreen wet forest (450 m a.s.l.). The individuals were shaken from trees during daytime or found sitting on understory vegetation at night.

### Sound registration and evaluation

The song was recorded in the laboratory (two males, CH7549-50 Collection Heller) using a digital bat-detector (Pettersson D1000X) with a sampling rate of 300 kHz and analysed using the program AMADEUS II (Martin Hairer; http://www.hairersoft.com). For frequency analysis in addition the programs CANARY (http://www.birds.cornell.edu/brp/) and ZeroCrossing v5 (kprestwi@holycross.edu) were used. The temporal pattern of the song was also analysed in a few recordings of low-frequency components, made with a SONY ECM-121 microphone connected to a personal computer through an external soundcard (Transit USB, “M-Audio”; 44.1 and 64 kilo-samples per second). Oscillograms of the songs were prepared using TURBOLAB (Bressner Technology, Germany). Most recordings (131 echemes) were made at temperatures between 18 and 21°C, a few (10 echemes) at 27°C were treated separately. The singers were caged in plastic tubes or gaze cages with microphone fixed or hand held at distances between 5 and 60 cm. One animal was also video-taped (without sound recording) while singing under weak red light. One complete song phrase is deposited at http://Orthoptera.SpeciesFile.org.

### Bioacoustical terminology

Syllable: sound produced during one cycle of movements (opening and closing of the tegmina); syllable period: time period from one syllable beginning to the next (reciprocal value: syllable repetition rate); echeme: first order assemblage of syllables; phrase: ± stereotypic combination of echemes and single syllables; pulse: undivided train of sound waves increasing in amplitude at the beginning and containing many similarly sized wave maxima and minima (cricket-like song structure); instantaneous frequency: cycle-to-cycle frequency based on zero crossing analysis

### Morphology

For measurements and images of the stridulatory file replicas were made using the cellulose nitrate technique (cellulose nitrate dissolved in acetone, put on the file and removed after drying; [18]).

## Results

**Figure 1:**
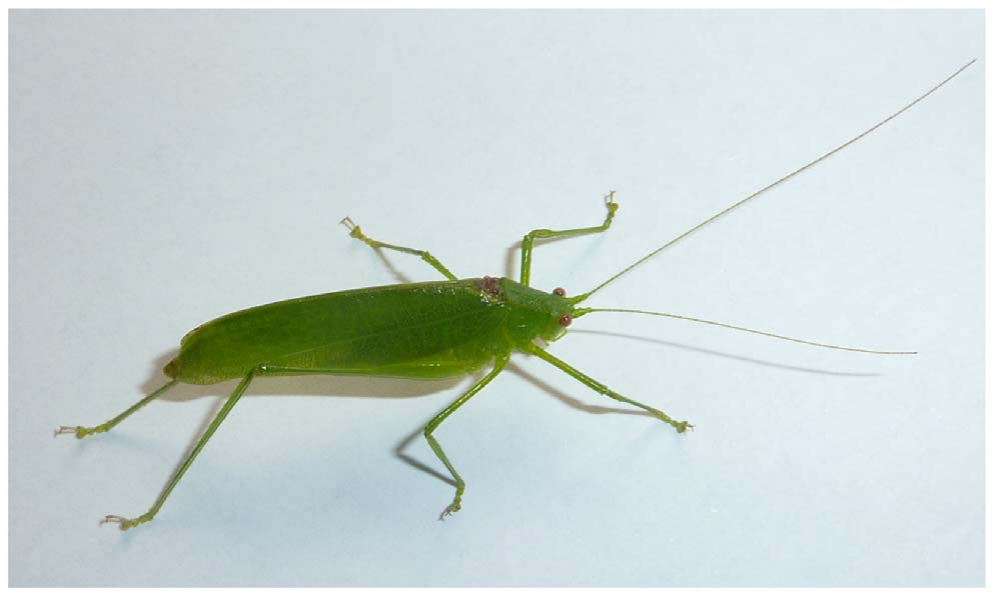
Habitus of a male *Ectomoptera nepicauda*, alive.

Males of *Ectomoptera nepicauda* are comparatively small, long-winged insects (Fig. 1; length of fore-wing 21–22 mm; n = 3), similar in size and general morphology to the wide-spread species *Phaneroptera falcata* (length of fore-wing 19–23 mm [19]). In captivity the two males were active only during darkness. Here they were observed to be very agile and flying easily even in small containers at least during some periods of time. Females have somewhat shortened and broadly rounded tegmina and are poor flyers due to their reduced wings.

### Morphology of sound producing organs

**Figure 2:**
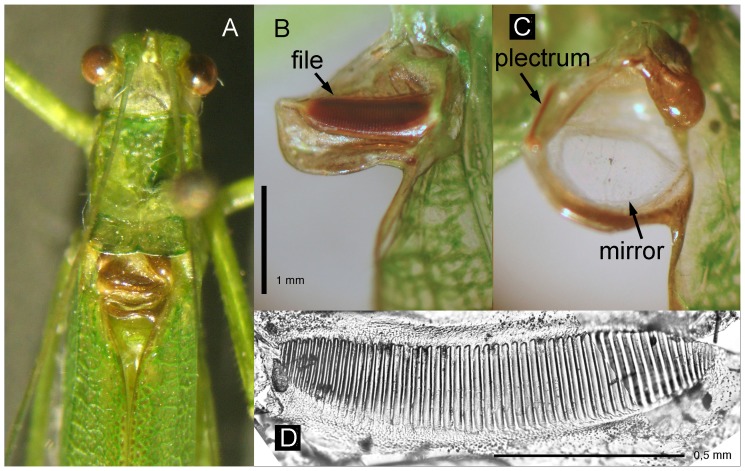
Acoustic parts of the tegmina. A Male from above, tegmina closed; B Left tegmen from below: stridulatory file; C Right tegmen from above: plectrum and mirror (same scale as B); D Stridulatory file.

**Figure 3:**
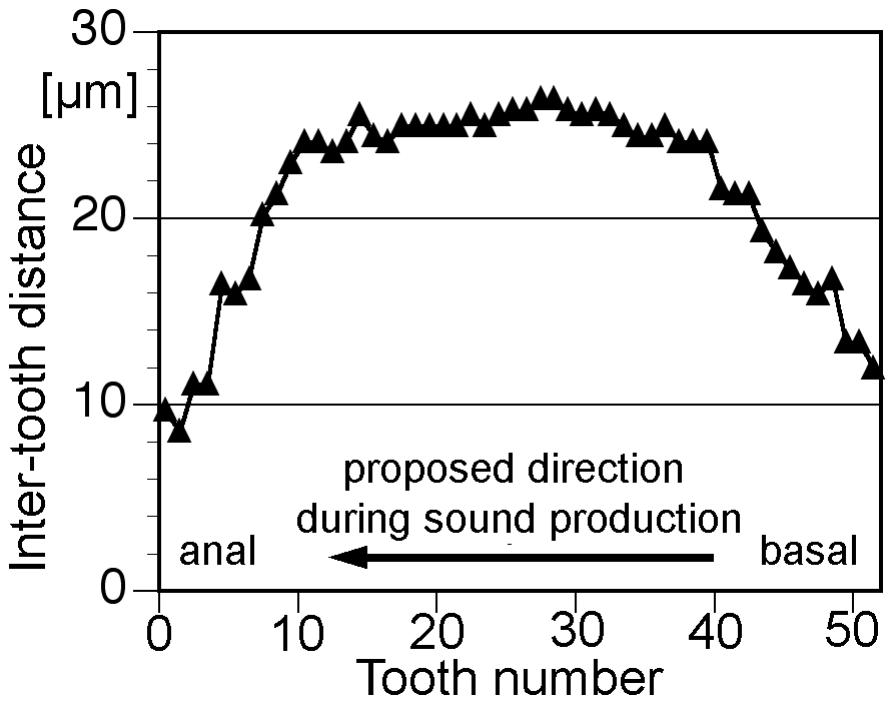
File structure. Intertooth intervals plotted in their natural sequence on the file.

The species got its generic name (Greek “cut away wing”) because the male fore-wings have “a deep excavation in hind margin just beyond stridulatory region, so the part of the metanotum is exposed even when fore wings are fixed” ([17]; Fig. 2A). This deep excavation is more conspicuous when the tegmina are spread (Fig. 2B). The dorsal part of the left tegmen consisted nearly exclusively of a strong stridulatory file with 54 broad teeth (n = 1; Fig. 2D): These teeth are spaced quite regularly over a large part of the file (Fig. 3; tooth density in the middle ca. 40 teeth/mm). The steep side of the teeth directs towards the wing base indicating sound production during the opening movement as described for *Phaneroptera* species [20]. The mirror is located on the right tegmen (Fig. 2C; diameter ca. 1 mm), slanted frontal-ward in an angle of about 30 degrees. Visible from above at the anterior-outside edge of the upper mirror frame a strongly sclerotized structure is visible even when the wings are closed.

### Song

**Figure 4:**
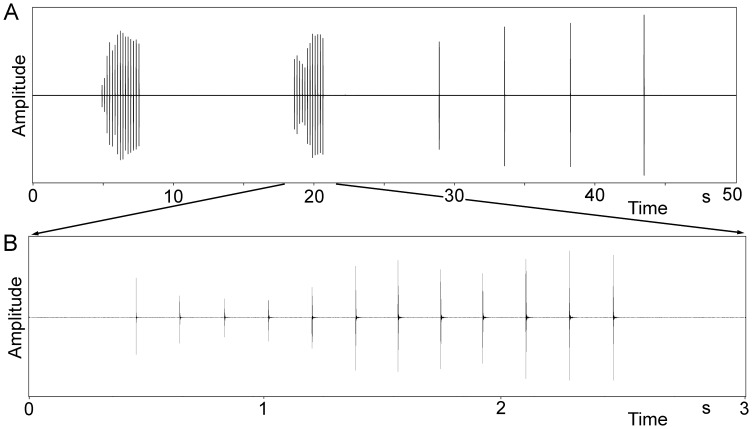
Calling song of *Ectomoptera nepicauda*. A Oscillogram of one complete phrase, containing two echemes and four single syllables. B Oscillogram of one echeme.

**Figure 5:**
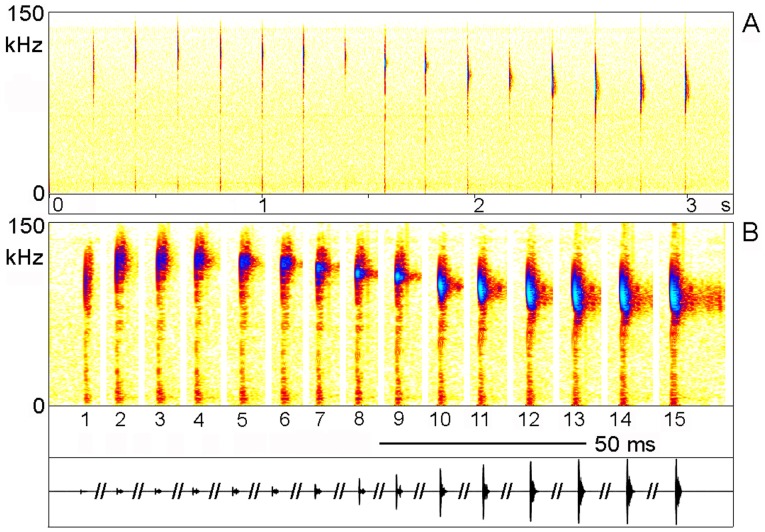
Calling song of *Ectomoptera nepicauda*. Sonogram of all syllables of one echeme. A Syllables and intervals in scale. B Intervals shortened, lowest track amplitude in scale.

**Figure 6:**
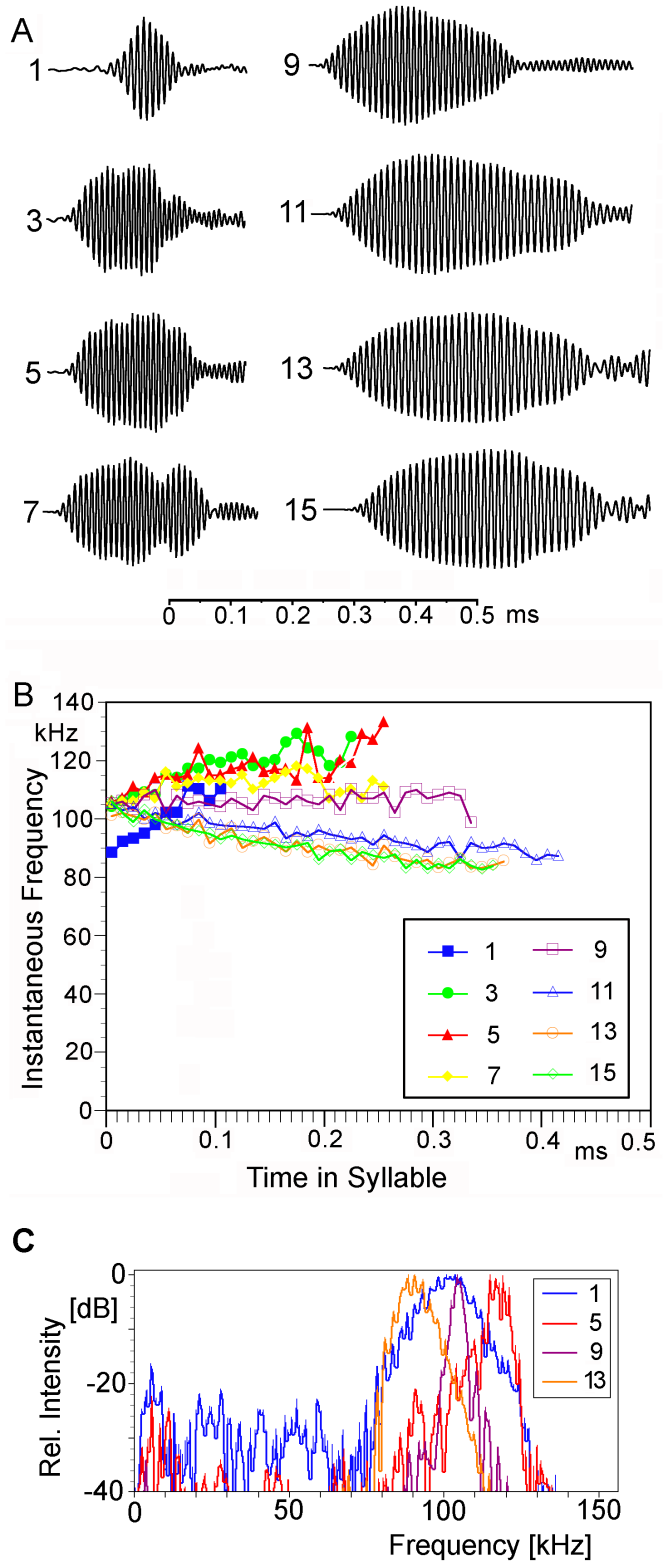
Calling song of *Ectomoptera nepicauda*. Oscillograms (A), frequency analysis based on zero-crossing (B) and power spectra (C) of selected syllables (see Fig. 5).

The calling song of the males (Fig. 4) consisted of long phrases, each containing one or two (mean 1.5; n = 131) relative densely packed series (echemes) of syllables which were followed by a sequence of syllables with much larger intervals (single syllables; n = 4.8±0,5; range 4–6; n = 31). The interval between the last syllable of the echeme and the first single syllable was always distinctly larger than the intervals between the single syllables (9.8±1.3 s compared to 5.1±0.4 s; n = 20/79; 10 phrases per male). These phrases had durations of 26 to 59 s (containing one echeme: 32.6±2.8 s; range 26–35 s; n = 9; two echemes 43.3±7.1; range 35–59 s; n = 20) and were repeated at intervals of several up to many minutes. From visual and video observation an alternative explanation that one echeme may result from one very slow wing movement can be excluded (see Supporting Information; Video S 1). In this case the wing would move over distances between 25 μm (jumping from one tooth to the next) and 0.2 mm (using the complete length of the file for seven pulses) for the production of one pulse, both hardly visible with naked eye or in the video. The echemes contained 7–24 syllables (1^st^ echeme: 13.2±3.2 syllables; range 8–24; n = 135; 2^nd^ echeme 11.6±3.5 syllables; range 7–21; n = 65) produced at a syllable repetition rate of 4.6 Hz (n = 272; 5 phrases per male; see Fig. 5a). In a few recordings made one month after the main study period and not evaluated otherwise (except for effects of temperature) the syllable repetition rate was 6.6 Hz at 27°C (n = 22). The duration of the syllables was extremely short and never exceeded 0.5 ms (highest number of wave periods counted 39–41). It increased from 0.1 to 0.4 ms within an echeme (Fig. 5, 6; see below), while the single syllables typically had the same duration as the last ones within an echeme, ca. 0.3–0.4 ms. Each syllable corresponds to one sound pulse as it is observed in the song of crickets (e.g., [2]). The duty cycle (percentage of time with active sound emission) measured within an echeme may thus be around 0.2% (13 syllables a 0.4 ms in 2.592 s) and within a phrase about 0.026% (30 syllables a 0.4 ms in 46 s).

To test if the males interact acoustically, we placed the containers with the animals closely together. The acoustical activity was relatively high with 50 registered phrases within two hours. In two cases the echemes overlapped, the second animal started immediately after the other male produced a second echeme. The echemes of both animals looked otherwise quite normal.

**Figure 7:**
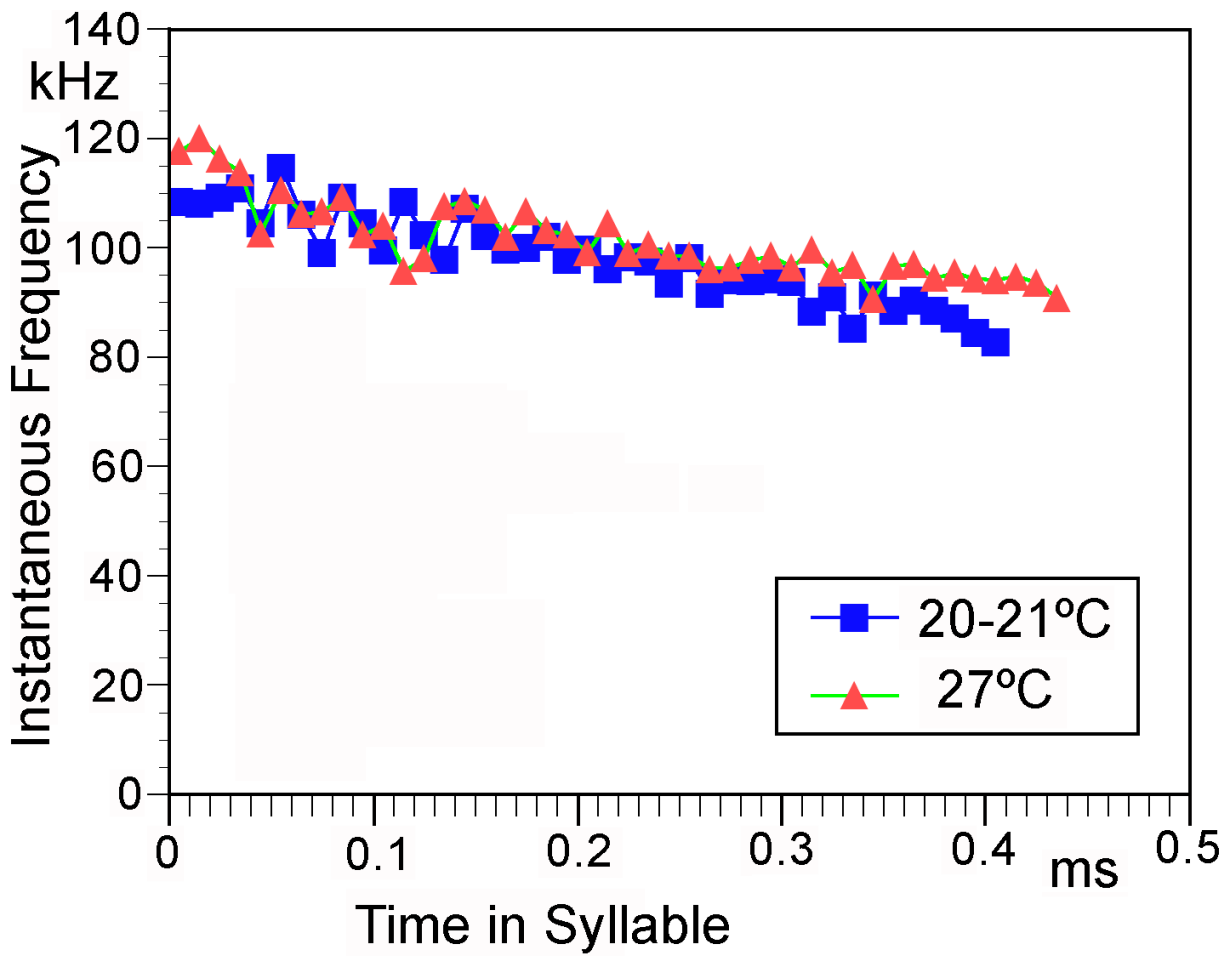
Effects of temperature on song. Instantaneous frequency based on zero-crossing analysis of single syllables at different temperatures.

The carrier frequencies within the syllables varied but were always quite high (Fig. 5). However, while the frequency composition can be easily recognised in the sonograms, the frequency change per time cannot be determined accurately using FFT spectral analysis for such short and frequency modulated songs. Similarly the quality factor Q does not provide much information about the resonator. Therefore we have used zero-crossing analysis (Fig. 6B). In addition, some power spectra are presented for comparison (Fig. 6C). The first pulse(s) were sometimes upward modulated sweeps from 80 to105 kHz. However, these distinctly upward modulated pulses were often missing. During the following pulses the steepness of the modulation decreased, but the end frequencies reached sometimes nearly 135 kHz, because the sweeps started at higher frequencies and got longer. After one syllable without distinct frequency modulation the pulses became downward modulated ending with loud and long sweeps from 105 to 80 kHz. The same frequency pattern was also observed in the single syllables. In the few recordings made at high air temperature the carrier frequency did not differ (Fig. 7; comparison of single syllables). Probably due to echoes (from the ground or container walls or the surrounding) the syllable structure was often not as regular as seen in the example shown in Fig. 6.

The speed by which the scraper touches the file teeth should be in the range of 2250 mm/s (calculated by assuming a carrier frequency of 90 kHz and tooth intervals of 0.025 mm).

## Discussion

### Morphology of the stridulatory organs

Taking symmetrical tegmina with file, plectrum and resonating areas on both of them as the plesiomorphic condition in the evolution of wing stridulation, the fore-wings of *Ectomoptera nepicauda* represent the most derived state among Ensifera. “Fiddling (finesse)”, the first word from the title of an important paper [21] about sound production in Phaneropterinae, might also be applied here, at least as the strict separation of driving and amplifying structures is concerned, comparable to string instruments. The fore-left wing carries only the file, the right one only plectrum and mirror. However, this situation seems not to be unique for this genus. There are at least two other phaneropterine genera and species with similarly built tegmina: Describing the South Indian species *Execholyrus alector* the author [22] gives a clear drawing of the structures (p. 100; Fig. 2) and wonders about the gap behind the acoustic parts. This species seems to be similar to *E. nepicauda* regarding structure of these parts and in their body dimensions. The left fore-wing has the same structure as in *Ectomoptera* also in the much larger *Macedna martini* Karsch, 1891 [23] from Sumatra, while the resonant parts of the right fore-wing are more complicated (see photos in [24]). In African species of the genus *Dithela* the left dorsal area is also reduced, but only to a size sufficient to protect the relatively large mirror ([17], photos in [24]). This gives an important clue for the understanding of the evolution of this extreme asymmetry. In general, the mirror on the right dorsal area is protected by the overlaying stiffer left tegmen. If the mirror is small – indicating frequencies in the ultrasonic range – then the left dorsal part can be reduced to the same small size as the mirror. Possibly in many phaneropterines this left dorsal field is really ‘mute’ (see Discussion in [6]) and has no function in sound amplification. In other bush-crickets, the situation may be different. In a species of *Panacanthus*, Montealegre-Z & Mason [11] measured some resonance of the left tegmen, and in some pseudophylline bush-crickets even a mirror-like structure can be found on the left side (e.g. in *Myopophyllum speciosum* Beier, a species with high-ultrasonic tonal song [25]; photos in [24]). So in Tettigonioidea there is not only a large diversity in size and shape of the acoustic parts of the tegmina, but probably also in their function.

As can be seen in Fig. 2C, the mirror is glassy and thin, a feature actually expected for non-resonant singers [26]). Its radius should have at least 1 / 6 of the carrier wavelength [27]. In *E. nepicauda* this size (radius 0.5 mm – calculated maximal wave-length 3 mm) corresponds only reasonably well with the observed wave-length (3.8 mm at 90 kHz).

### Song

In time pattern, containing one type of syllables, but several types of intervals with one phrase extending over quite a long period of time, the song of *Ectomoptera nepicauda* certainly belongs to the more complicated songs among Phaneropterinae. However, its most remarkable features are found in the frequency domain. Firstly, the carrier frequencies are situated in the high ultrasonic range, around 100 kHz. This has not been observed in any other phaneropterine and it is rare also in other tettigonioids (see [25], [28]). Secondly, each syllable consists of only one cricket-like, frequency-modulated pulse. In a typical phaneropterine species, a syllable would consist of a series of heavily damped impulses each produced by one contact of a file tooth with the plectrum. All other species with tonal songs in this frequency range use short spaced tonal pulses (SSTP) [25]; during one sound producing movement the plectrum performs some short runs over the file thus producing several pulses. This pattern is assumed to be based on special properties of the plectrum which has a complicated structure and should contain elastic elements for storing energy [25]. The song of *Ectomoptera*, however, demonstrates that a SSTP structure is not necessary for high-frequency tonal songs. Obviously here the animals can achieve a very high file-to-scraper-speed, higher than in any species studied [25], during the whole movement. The syllables in the song of *Eurycoyrpha resonans* Hemp show the same one-pulse-structure but with much lower carrier frequency [14]. Future studies have to show if for the production of such songs elastic structures are involved and where they might be localised. An additional hint to the involvement of such structures comes from the observation that with changing temperature the syllable repetition rate changes, but not the frequency of the song (Fig. 7). Unfortunately our data are too limited to draw any safe conclusions. The situation becomes even more complicated if the song is produced during the opening movement as suggested by the tooth structure. Since in most, but not all (see [28]) other high-frequency singers (and most tettigonioid species studied) the sound is produced during wing-closure the elastic elements would have to be used in the opposite direction.

The frequency modulation within the pulses may result from differences in the speed of the movement of the tegmina (see also [29]). Probably the driving force declines during the movement with the downward modulated loud pulses at the end of an echeme and with single syllables. For the soft upward modulated pulses or pulses without frequency modulation at the beginning some kind of increasing activation of the muscles has to be assumed.

### Communication

To understand the function of this unusual song one has to have the properties of the communication system of phaneropterines in mind. In this family the females typically respond acoustically to the male song [30]. This is very likely also true for *Ectomoptera nepicauda*: the females have well developed stridulatory organs (own observations), but are equipped with broad tegmina certainly much less adapted for flying than the males. So the male has not necessarily to sing and to wait for a responding female. For him, being able to fly, it may be a better strategy to move singing through the habitat and signal at many different places.

If the female response had a lower carrier frequency than the male song – as can be expected from the typical female phaneropterine tegmina in this species - then the situation would be similar to that in *Holochlora nigrotympana* Ingrisch, a Thai phaneropterine. Here the female signals with lower frequencies and is heard over a larger distance than the ultrasound producing male [31]. Also in *Ectomoptera* the range of the song may be quite restricted due to strong attenuation of ultrasound especially in humid air (see Discussion in [32]). However, when emitting his song the male simultaneously uses another communication channel: by producing the extremely short syllables with very high movement speed, he generates vibrations. These ground vibrations are so strong that they can be picked up by unaided ears and audio-microphones if the singer sits on suitable ground (own observations). They could replace the separate tremulatory signals (vibrations without air-borne sound) used by neotropical pseudophylline bush-crickets [32], [33]) and may also be answered by the female.

About the ultimate reasons for the use of high ultrasound and of pure tones versus broad banded signals can only be speculated (see [25], [26]). In any case, however, the song of *E. nepicauda* shows that tonal songs are not necessarily narrow-banded sounds restricted to one particular frequency.

## Supporting Information

Video S1 **Male of** ***Ectomoptera nepicauda*** **stridulating (without sound).**
(MOV)
